# Unlocking cardiac motion: assessing software and machine learning for single-cell and cardioid kinematic insights

**DOI:** 10.1038/s41598-024-52081-9

**Published:** 2024-01-20

**Authors:** Margherita Burattini, Francesco Paolo Lo Muzio, Mirko Hu, Flavia Bonalumi, Stefano Rossi, Christina Pagiatakis, Nicolò Salvarani, Lorenzo Fassina, Giovanni Battista Luciani, Michele Miragoli

**Affiliations:** 1 Department of Surgery, Dentistry and Maternity, University of Verona, Verona, Italy; 2 Department of Medicine and Surgery, University of Parma, Parma, Italy; 3 Deutsches Herzzentrum Der Charité, Department of Cardiology, Angiology and Intensive Care Medicine, Berlin, Germany; 4 Humanitas Research Hospital, IRCCS, Rozzano (Milan), Italy; 5 Department of Biotechnology and Life Sciences, University of Insubria, Varese, Italy; 6 Institute of Genetic and Biomedical Research (IRGB), UOS of Milan, National Research Council of Italy, Milan, Italy; 7 Department of Electrical, Computer and Biomedical Engineering, University of Pavia, Pavia, Italy

**Keywords:** Image processing, Machine learning, Software, Stem cells, Cardiology, Biomedical engineering

## Abstract

The heart coordinates its functional parameters for optimal beat-to-beat mechanical activity. Reliable detection and quantification of these parameters still represent a hot topic in cardiovascular research. Nowadays, computer vision allows the development of open-source algorithms to measure cellular kinematics. However, the analysis software can vary based on analyzed specimens. In this study, we compared different software performances in *in-silico* model, *in-vitro* mouse adult ventricular cardiomyocytes and cardioids. We acquired *in-vitro* high-resolution videos during suprathreshold stimulation at 0.5-1-2 Hz, adapting the protocol for the cardioids. Moreover, we exposed the samples to inotropic and depolarizing substances. We analyzed *in-silico* and *in-vitro* videos by (i) MUSCLEMOTION, the gold standard among open-source software; (ii) CONTRACTIONWAVE, a recently developed tracking software; and (iii) ViKiE, an in-house customized video kinematic evaluation software. We enriched the study with three machine-learning algorithms to test the robustness of the motion-tracking approaches. Our results revealed that all software produced comparable estimations of cardiac mechanical parameters. For instance, in cardioids, beat duration measurements at 0.5 Hz were 1053.58 ms (MUSCLEMOTION), 1043.59 ms (CONTRACTIONWAVE), and 937.11 ms (ViKiE). ViKiE exhibited higher sensitivity in exposed samples due to its localized kinematic analysis, while MUSCLEMOTION and CONTRACTIONWAVE offered temporal correlation, combining global assessment with time-efficient analysis. Finally, machine learning reveals greater accuracy when trained with MUSCLEMOTION dataset in comparison with the other software (accuracy > 83%). In conclusion, our findings provide valuable insights for the accurate selection and integration of software tools into the kinematic analysis pipeline, tailored to the experimental protocol.

## Introduction

The beating heart is the result of optimal electromechanical synchronization. Due to the high interconnection between electrical and mechanical activities, researchers developed multiple types of models trying to unveil and quantify both from cellular to whole organ levels^1–5^. Extensive and exhaustive studies have been performed on the electrical counterpart in both physiological and pathological conditions, starting from single cells and organ slices^6–8^, and broadening to *ex-vivo*^9^ and *in-vivo* models^10,11^. Thanks to the recently improved computation ability, both heart functions were studied with *in-silico* simulations, as an alternative to animal experimentation^12–14^. When purely measuring the mechanical activity of the heart, *in-vitro* studies face multiple challenges compared to *ex-vivo* and *in-vivo*. Specifically, kinematics and contraction force measured *in-vitro* need to address the micrometric cell dimension and forces expressed and require specific and expensive instrumentation^15^. The advent of computer vision technologies in biology brought alternative solutions to cell movement tracking and analysis^16,17^. In detail, motion tracking analysis aided by high-temporal and spatial resolution cameras has been capable of recording the whole kinematic evaluation^18–22^. Traction force microscopy is an expression of this computer vision approach, which has the seeding of the cells on materials with well-known mechanical properties to indirectly estimate the force of contraction as a counterside^23–28^. In the case of samples with weak or even without attachment points, the benchmark in vision-aided motion analysis has been the IonOptix^®^ system, for over two decades^29–31^. This software can evaluate the micrometric sarcomere shortening in single isolated cells and recently expanded to movement in cardiomyocyte (CM) layers derived from human induced pluripotent stem cells (hiPSCs-CM)^29,32^. Many other algorithms based on information extracted from video frames^33,34^, were implemented to develop open-source, highly flexible, and user-friendly software capable of estimating the kinematics from video recordings, while enabling researchers to tailor the code to their experimental needs^35^. Noteworthy, MUSCLEMOTION (MM)^36^ has set itself as one of the most used open-source software based on image information (e.g., image intensity-based segmentation and tracking). Moreover, open-source solutions help to evaluate kinematics in custom high-throughput platforms with advanced image processing such as optical flow algorithms^37,38^. Among many tracking algorithms, which base their computation on this concept, one worth mentioning is CONTRACTIONWAVE (CW) validated in the recent work from Scalzo et al.^39^. The study produced a user-friendly, python-based interface able to analyze in parallel multiple videos and it was validated with both single cells and hiPSCs-CM 2D layers. Lastly, other notable solutions are the use of algorithms based on marker-aided tracing derived by the detection of blob objects with different shapes and dimensions^40,41^. This analysis is mainly based on the hypothesis of rigid blob movement, which is a valid assumption for rod-shaped cells with rapid 2D shortening as well as 3D *in-vitro* models such as the attachment points of engineered heart tissue (EHT)^21,42^. One method that demonstrated both consistency and flexibility is the Video Kinematic Evaluation (ViKiE). In this software, the analysis begins by tracking the marker trajectories which are then analyzed by a customizable MATLAB^®^ code to retrieve kinematic parameters either *in-vitro* or *in-vivo* at the preclinical and clinical level^18,43–47^.

The performance of the listed software varies depending on the samples analyzed^48^. Specifically, the *in-vitro* solutions range from adult and neonatal CMs isolated from the tissues to hiPSCs-CM 2D layers and 3D constructs^49^. One of the biological models driving the interest of scientists is represented by cardioids, which can be highly reproducible and provide the chance to make a high-throughput study yet limited by fetal-like gene expression^50^. The cardioids are normally formed by CMs, differentiated from hiPSCs, and demonstrated to resemble *in-vitro* the organ-like organization more than expected^51,52^. Moreover, hiPSCs-CM were shown to express caffeine-responsive stores for sarcoplasmic reticulum Ca^2+^, offering the opportunity to study their response with well-known inotropic substances^53^.

The relevance of the models described, mixed to an engineering approach, brought to a faster and more efficient elaboration of the information, with high potential and flexibility across different fields^54–58^.In fact, the use of machine learning (ML) in biology and medicine allowed to unveil correlations and phenomena hidden in the vast amount of data, outperforming the analysis of experts in the field, for example in the interpretation of the electrocardiogram^59–63^. Supervision is based on previously labeled reference datasets which are used to train ML algorithms to predict the class of each unlabeled data point^58^. Classification has already been used in the in-vitro context for several tasks, for instance, to check the quality of CM derived from differentiated human iPS cells^64 ^or to identify healthy or diseased CM from the contractile profile^64^. ML algorithms, being able to consider the complete set of variables and making an automatic cluster detection in the collected data, allowed a significant improvement both in drug screening and prediction^65^.

This work compares three different approaches to evaluate contraction kinematics in both ventricular isolated single cells and cardioids. The kinematic analyses are performed with the three computer vision methods mentioned above: MUSCLEMOTION (MM), CONTRACTIONWAVE (CW) and Video Kinematic Evaluation (ViKiE). MM is considered as the benchmark, already extensively validated^36^. The main goal of the study is to evaluate and compare the applicability of these software programs on single cells and cardioids. Finally, we implemented ML algorithms to test the prediction performance of the three training data sets generated from each software and their overall sensibility in eventual kinematic changes following the administration of inotropic substances.

## Methods

### Open-source software algorithms and data analysis

The first selected algorithm was MUSCLEMOTION (MM)^36^: a Fiji^®^-implemented macro for motion tracking. The cell contraction is evaluated as the mean pixel intensity of the image resulting from the difference between two frames^36^. Thus, this tracking approach relies on high quality and sharp contrast of the images to get correct measure. The binarization of the images allows the calculation of the maximum displacement over time as the difference in intensity between a reference image and the other frames. From the contraction profile, the velocity is derived by the first derivation of this profile. According to its nature, MM can report the average kinematics of the sample, highly dependable on the quality of image intensity and resolution, with the ability to perform a single pixel movement evaluation, as stated in the work of Sala et al.^36^.

The second software was CONTRACTIONWAVE (CW)^39^. This algorithm is Python-based with the possibility to process multiple videos or sequences of images at once. The tracking software relies on a dense optical flow algorithm to quantify the speed of contraction profiles^39^. Briefly, the algorithm evaluates the vectorial contraction field between successive frames which is corrected for the image dimension and frame-per-second returning the speed profile. Furthermore, this software enables the user to save all detected waves in each video, along with metrics such as beat duration, time to peak of contraction, and time to peak of relaxation. The software leverages the optical flow principle and evaluates the displacement field using all pixels of the image^39^.

The third software was Video Kinematic Evaluation (ViKiE) which is a pipeline consisting of an open-source tracking software (Video Spot Tracker, version 08.11, CISMM) and a custom MATLAB^®^ script used in motion analysis^18^. The software Video Spot Tracker (VST) can track the sample motion using an appropriate marker as in feature-based tracking systems. The marker is hereby defined as appropriate when it correctly detects and follows the sample movement. Different kernels are available in the software and the best choice is user dependent as well as where the marker is positioned. The tracking output is influenced not only by the kernel of the tracker but also by its dimension, which defines how extended the sample-region tracked is. Once the user has obtained the optimal setups, the VST software output is pipelined with a MATLAB^®^ script to process the coordinates calculated in the reference system of the image. In the analysis, the origin of the reference system is set on the upper-left corner of the first video frame. Thus, the contraction profile is derived from the sequence of coordinates acquired with VST, whilst the velocity is retrieved by the first derivation of the marker positions corrected by the pixel dimension. As in CW, an updated feature of the ViKiE software allows the user to indicate the starting and ending point for each beat to calculate the duration, as well as the time to reach the peak of both contraction and relaxation phases on the speed profile.

**Figure 1:**
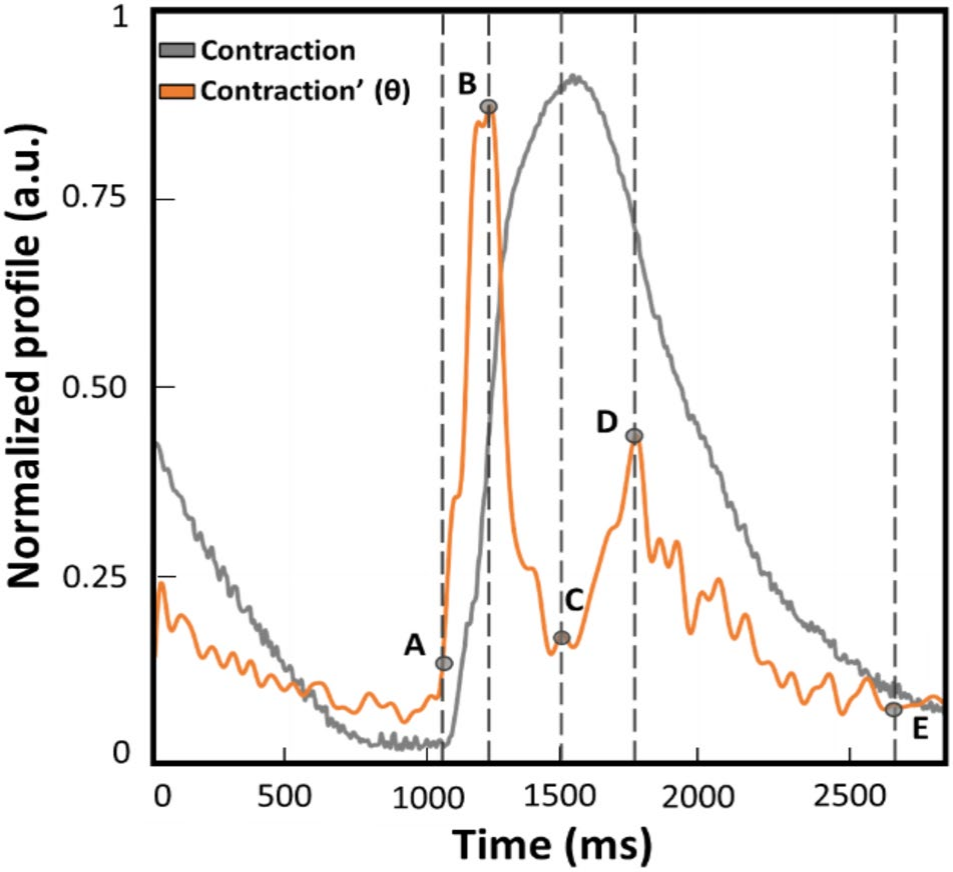
Example of normalized contraction profile (grey trace) and its first derivative named θ (orange trace) obtained with MUSCLEMOTION (benchmark). This represents a qualitative graphical description of the kinematic parameters calculated and compared during the experiments. The time between points A and E is defined as beat duration. The time between points B and D is the distance between the peaks of contraction and relaxation (t[θc−r]). The time between points A and B corresponds to the interval of maximum speed during the contraction phase (t[θc]). Lastly, the time between C and D is defined as the interval of maximum speed during the relaxation phase (t[θr]).

Once the raw data were acquired during the experiments described in detail below, the post-processing of the kinematics was performed with the listed open-source programs and the absolute values of speed profiles were compared with the parameters displayed graphically in Fig. 1.

All the videos were recorded with consistent frame rate throughout the experiments and in the same conditions. Moreover, it has to be noted that the profiles reported throughout the work will be indicated as absolute values to avoid misinterpretation and ensure consistency in data representation of the outputs from the three computer vision software. From the absolute of the speed profile, four parameters were calculated to assess any difference in the kinematic evaluation. A representative contraction profile and its first derivate (named as θ) are displayed in Fig. 1 to highlight the parameters calculated on each different software output. The profiles reported were derived from the benchmark MM and normalized. A remark from the authors is the slight temporal anticipation of the speed profile due to the approximation of the ratio of increments from which the first derivate is calculated. As illustrated in Fig. 1, the parameters over the speed profile (orange trace) were selected according to the physiological contraction response (grey profile). The difference between points E and A is defined as beat duration and corresponds to the time elapsing between the 5% of the end of contraction and the onset defined as the first value different from zero. The difference between points B and A is the time required to reach the maximum speed of the contraction phase (t[θ_c_]), as the maximum slope in the ascending phase of the contraction profile. The difference between D and C is the maximum speed reached during the relaxation phase (t[θ_r_]), namely the maximum slope in the descending phase of the contraction. The distance between points D and B is considered the distance between the peaks of contraction and of relaxation (t[θ_c-r_]).

### In-silico cardiomyocytes

By kind courtesy of Prof. Sala, a batch of the *in-silico* cardiomyocyte (CM) videos, used to establish the MM method, was employed to perform the first comparison between the three software. Briefly, the videos were created using Blender software (v2.77, Stichting Blender Foundation) to simulate cardiomyocyte-shaped objects in motion. The *in-silico* simulations were designed with two different graphical patterns: (i) a grey-based with repetitive black bands present along the structure in both longitudinal and perpendicular directions and (ii) a diffuse phase contrast to simulate highly repetitive pattern of an *in-vitro* single CM^36^. The videos were analyzed with two different time-to-peak values (50% and 200% of the baseline value considered 100%) but with constant amplitude of contraction. The software programs were evaluated based on their capability to estimate the correct kinematic stated by MM. Moreover, both qualitative and quantitative comparison of the output parameters was performed over a sample beat, as described above.

### Ventricular cardiomyocytes isolation

Single ventricular cells were derived from 6-month-old mice via Langendorff isolation (Louch et al., 2011). All experiments were performed according to the 2010/63/EU Directive and approved by the ethics committee of Humanitas Research Hospital, with code 07/2019. Moreover, the animal experimentation was performed in accordance with the ARRIVE guidelines, and all the authors complied to the above regulations. Briefly, mice were anesthetized with ketamine (100 mg/kg) and xylazine (10 mg/kg) administered via intraperitoneal injection. Mice were placed in dorsal recumbency, and after chest opening, the heart was excised. The aorta was cannulated with a 21-gauge needle and secured to a Langendorff apparatus. Cardiomyocytes were obtained by enzymatic perfusion of the left ventricle with Collagenase Type II (Worthington, LS004177, ≥ 125 units per mg). Hanks Balanced Salt Solution (HBSS 1x, Invitrogen 14,170–088) supplemented with magnesium chloride hexahydrate (1 mmol/L, Merck 442,611), taurine (30 mmol/L, Merck T0625), D-( +)-glucose (15 mmol/L, Merck G5767), magnesium sulfate (1.2 mmol/L, Merck M2643) was made to further process the cells. The digested heart was removed from the apparatus and submerged in stop solution (4% Bovine Serum Albumin, BSA, in supplemented HBSS), and the left ventricle was dissected from the right ventricle and atria. The tissue was gently minced and pipetted to release cells. The cells were filtered through a 100 µm-mesh strainer to avoid contamination with undigested tissue fragments. CMs were sedimented by centrifugation at 120xg for 30 s. The cell pellet was washed 3 times in supplemented HBSS and brought at the correct calcium concentration with gradual additions. Briefly, 10 µL from the stock solution (10 mmol/L of CaCl_2_ in HBSS) were added in two steps, and a last dilution of 10 µL ultimate the calcium addition, bringing the cells to a final extracellular concentration of 2 µM. The single cell dimensions were measured via Fiji^®^.

### Frequency protocol and single-cell data acquisition

The cell suspension from the tissue isolation was placed on a heated perfusion chamber at 37 °C equipped with silver wires for field stimulation. Cell pacing was generated using the Myopacer Field Stimulator from IonOptix^®^ at 40 V cathodal stimulation. Once settled at the bottom of the chamber, the cells underwent a frequency protocol at the following steps: 0.5 Hz, 1 Hz and 2 Hz; 5 s videos of paced cardiomyocytes were acquired by a high-speed camera (Basler, acA1300-200 um) at 143 fps, using PylonViewer 5 software (Basler, version 5.1.0.12681 64-bit) and an image dimension of 896 pixels × 980 pixels. The hardware used for the acquisition mounted an Intel(R)^®^ CoreTM i7-8750H CPU at 2.20 GHz, RAM 16 GB on Windows 11 Pro, version 22H2. The cells that respected the entire protocol of pacing were analyzed.

### Stem cells culture and 3D cardioid formation

Human embryonic stem cells (RUES) were kindly provided by Dr. Elisa Di Pasquale. The stem cells (passage < 30) were seeded on Matrigel^®^-coated well plates in Essential-8 medium (Gibco™, #A1517001) and, once reached 70–80% of confluency within 3 to 4 days, differentiated into cardiomyocytes. The differentiation was performed via the PSC Cardiomyocyte Differentiation Kit (Gibco™, #A2921201). Briefly, the protocol induced differentiation through sequential refreshment of the three different media in the kit every two days (medium A, B and M). Once in medium M, the cells were refreshed every 2 days. On the 26th day of culture, the cells were purified from all non-cardiomyocytes by MACS PSC-Derived Cardiomyocyte Isolation Kit (Miltenyi Biotec, #130-110-188) as the negative fraction to depletion antibodies. A resuspension of 50.000 cells in DMEM (Gibco™, 11960-044 supplemented with glutamine 1:1000) completed with 10% FBS (Microgem, S1860-500) and 1% P/S (Euroclone, ECB3001D) was seeded into round-bottomed ultra-low attachment 96-well plate. After 4 days, the cells started to form small clusters and within a week began to compact into cardioids (Hofbauer et al., 2021). The medium was partially refreshed every 3 days. The cardioids dimensions were measured via Fiji®.

### Frequency protocol and cardioid data acquisition

At day 50 of culture, the cardioids were singularly picked and transferred into a field stimulation chamber (Warner instruments, RC-49MFSH) at 37 °C and 5% CO_2_, powered by the stimulus generator STG4004-16 mA (Multi Channel Systems MCS GmbH) at 8 V with a step cathodic function, duty cycle 20 ms, and increasing frequencies of 0.5 Hz, 0.75 Hz and 1 Hz. From previous experiments, we observed that the maximum pacing frequency to keep the 1:1 ratio was 1 Hz, leading to an adaptation of the frequency protocol employed in the single cells. Videos of 10 s at 143 fps were recorded using a high-speed camera (Basler, acA1920-155 um), driven by PylonViewer software 6 (Basler, version 6.2.0.8205), running on an Intel® CoreTM i5-9400F, CPU at 2.90 GHz, RAM 16.0 GB, Windows 11 Home, version 21H2, grabbed image dimension of 1000 pixels × 1000 pixels. For each condition, a biological triplicate was assured.

### Caffeine and potassium treatments

To assess software sensibility, two substances were added to the sample medium during the frequency protocol described in detail above. Specifically, samples underwent either a controlled increase of extracellular potassium for inducing depolarization (KCl, Sigma-Aldrich P9541) or an addition of caffeine (Sigma-Aldrich W222402) at 10 mmol/L concentration for inducing inotropic response. These concentrations were assessed to be the most effective kinematic-wise based on previous experiments and the literature^66,67^. Five minutes after the substances administration, videos were recorded and collected for further data analyses, as previously described.

### Dataset and supervised machine learning

For each algorithm (MM, CW, and ViKiE), we produced a dataset at each frequency of stimulation in either adult single cells or cardioids for a total of 18 datasets. The treatment factor was considered to be either 10 mmol/L caffeine or 10 mmol/L KCl. The datasets consisted of the 4 kinematic measures (Fig. 1), two time-dependent parameters (the contraction and relaxation peaks magnitude), and the classification label (treated or control). Finally, the rows contained the parameters of each beat. We added the contraction and relaxation peaks magnitude to the set of parameters to have a larger number of features for the classification. The datasets were split into training and testing sets with a ratio of 0.80. In the case of an unbalanced dataset, i.e., composed of an unequal amount of data in one class compared to the other (treated vs control), a re-balancing by sampling a subset of the overrepresented class was made and merged with the underrepresented set.

Machine learning was applied by using random forest (RF) and support vector machine (SVM), the latter with two different kernels (linear and polynomial). RF algorithm was selected because it emerged as the best approach in a study classifying contractile profiles^64^, whereas the SVM algorithms were selected as commonly used in machine learning. Specifically, SVM with linear and polynomial kernels obtained the highest performance scores. SVM algorithms are classification tools based on four basic concepts: the separating hyperplane, the maximum-margin hyperplane, the soft margin, and the kernel function. The first three elements help to select the optimal hyperplane separating the data points to be classified^68^, while the kernel function modifies the distribution of the data on the coordinate system to better separate them. RF algorithm is based on an ensemble of decision trees, each working on randomly selected features and a sample of data extracted from the training set^69^. All the decision trees in the RF perform a classification and, in the end, a majority vote is pooled. The described algorithms were employed to classify the datasets described above. The performance metrics considered in this work were the true positive rate (TPR) (Eq. (1)) and accuracy (Eq. (2)), which are defined according to the values of the confusion matrix, as shown in Table 1.

1 $${\text{TPR}} = {\text{TP}}/\left( {{\text{TP}} + {\text{FN}}} \right)$$

2 $${\text{Accuracy}} = \left( {{\text{TP}} + {\text{TN}}} \right)/\left( {{\text{TP}} + {\text{TN}} + {\text{FP}} + {\text{FN}}} \right).$$

**Table 1:** Example of confusion matrix.

	True values		
Predicted values		+ (1)	− (0)
	+ (1)	TP	FP
	− (0)	FN	TN

*TP* true positive, *FP* false positive, *FN* false negative, *TN* true negative.

### Ethics approval

The mice adult ventricular cardiomyocytes were obtained in accordance to the protocol code 07/2019, approved by the ethical committee Directive of Humanitas Research Hospital and according the 2010/63/EU Directive.

## Statistics

Statistical analysis was performed using GraphPad Prism software (version 8.0.2). Once checked the normal distribution of the data with the Kolmogorov–Smirnov test, a two-way ANOVA was performed, correlated with the Tukey test for multiple comparisons. The treatment was considered as “within”-samples factor while the technique as “between”-samples factor. When specified, supplementary two-way ANOVA was performed to test the effect of the pacing protocol considering the frequency as “within”-samples factor. In this case, the “between”-factor was defined as the software employed during the analysis. Again, the Tukey test was applied for multiple comparisons. Biological triplicates were assured, except when specified due to analysis issues. In the text and the figure, data are reported as mean ± standard deviation and significance was accepted when p < 0.05.

## Results

### In-silico results

**Figure 2:**
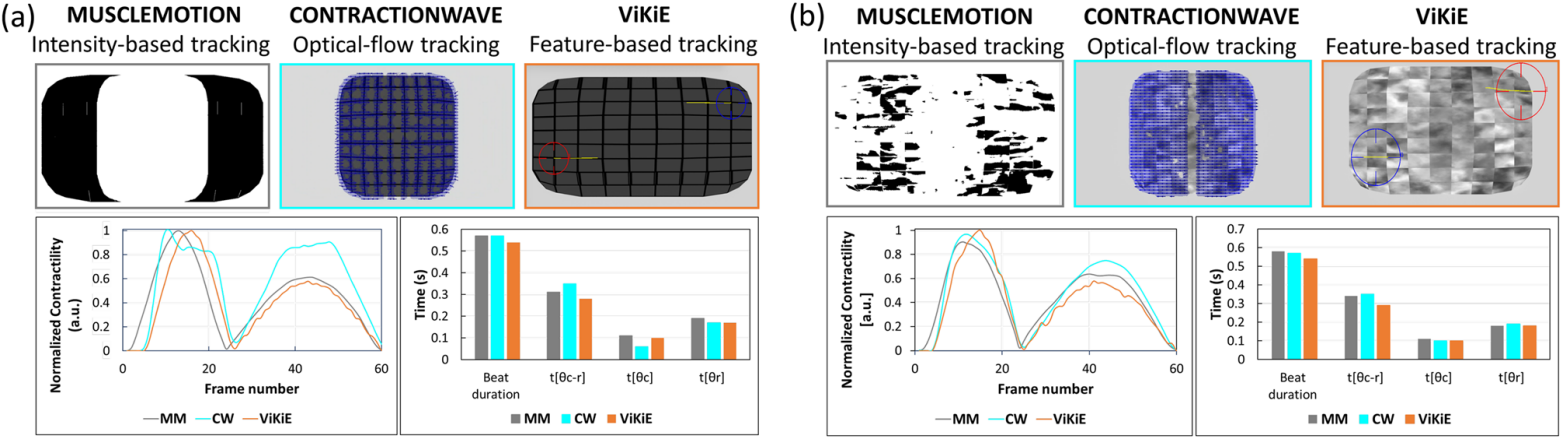
Comparison of the three open-source software programs with *in-silico* cardiomyocytes with 200% time-to-peak. (**a**) The first experiment with “grey” patterned cardiomyocyte. The plot on the left side of the panel represents the speed profile evaluated by the three software programs. The grey profile is the benchmark considered in this study (MUSCLEMOTION), the cyan profile derives from CONTRACTIONWAVE evaluation, and the orange profile is the ViKiE estimation. The histograms on the right side represent the four parameters extracted by the profiles and measured by the three methodologies. (**b**) The second experiment with “fog” patterned cardiomyocyte. As in panel (**a**), the profiles and histograms of the software evaluations are reported to address the ground-truth comparison.

The three software programs were first compared with *in-silico* simulations. The raw data were derived by modification of the time-to-peak values, namely 50% (Tvar50) and 200% (Tvar200) of baseline. Figure 2 reports the performance of the algorithms over the Tvar200 experimental condition. An introductive qualitative comment can be made on the profiles reported in the lower-left part of Fig. 2a. Overall, the main difference of the profiles resides on the kurtosis of the peaks.

It is trivial to observe that CW is more dispersed over the peaks in comparison to the other two software programs in this condition. The histogram of the same panel confirms that, although CW correctly evaluates the beat duration, it poorly performs in estimating the t[θ_c_] as well as t[θ_r_]. In the latter parameter it lines up with ViKiE measurement. Observing the quality of the profiles in Fig. 2b, all three software programs converge to the same shape, with some fluctuations in the ViKiE profile. This condition, which resembles the physiological condition, leads to more consistent evaluations of CW and ViKiE in comparison to MM. The illustrated profiles and histogram from the Tvar50 video can be found in Fig. S1.

### Single cell kinematic assessment

**Figure 3:**
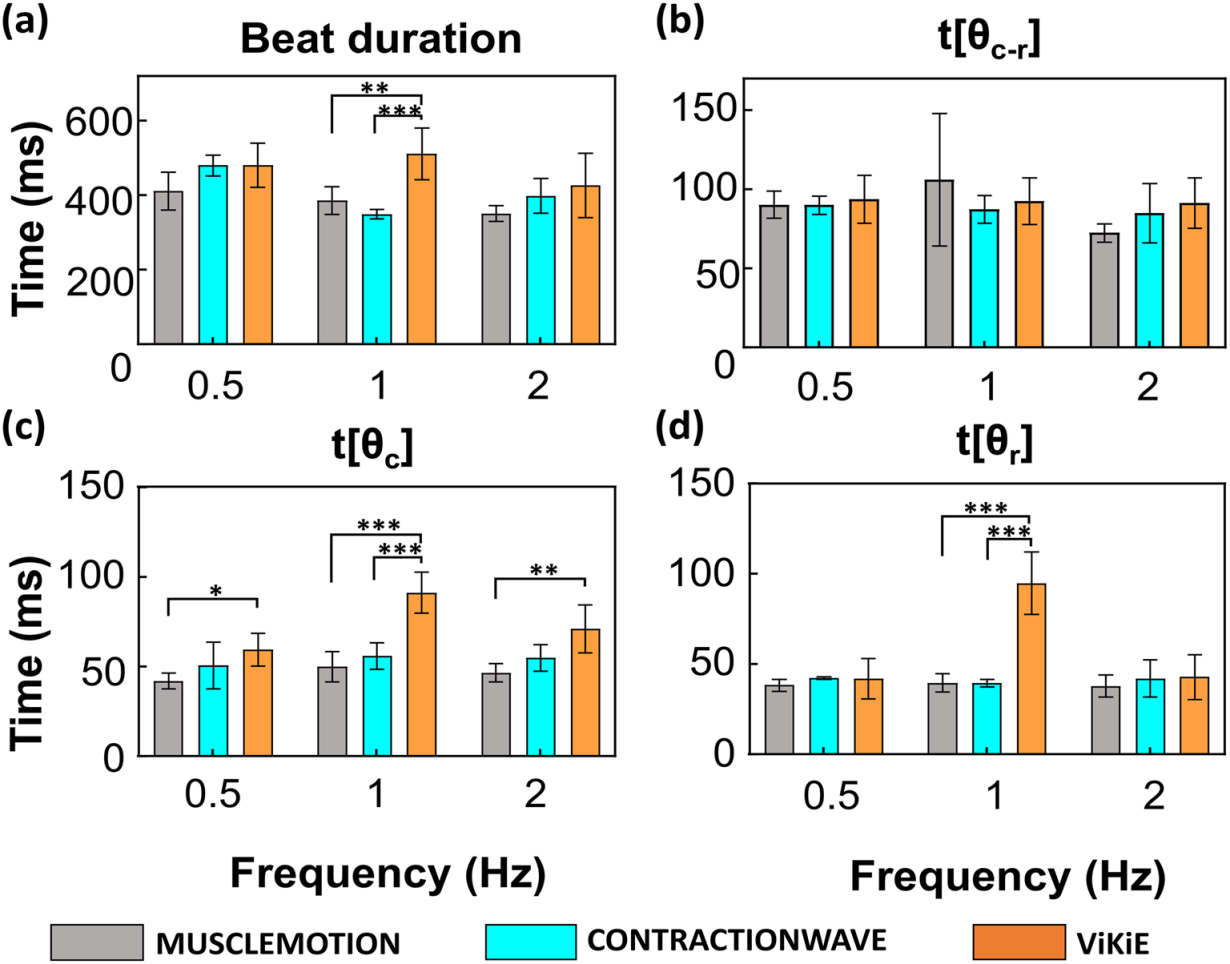
The frequency response of single cells was measured with the three open-source software programs. Grey: MM, Cyan: CW, Orange: ViKiE. (**a**) Beat duration of the untreated cells, for each algorithm. X-axis: frequency of stimulation in Hz (s^−1^). Y-axis: time occurred in milliseconds (ms) (**b**) The estimation of the time occurring between the double speed peaks. (**c**) The maximum speed reached during the contraction phase is reported in this panel. (**d**) The maximum speed reached during the relaxation phase t[θr]. Two-way ANOVA was performed with Tukey statistical hypothesis for multiple comparisons, with significance levels defined *p < 0.05, **p < 0.01, ***p < 0.001 (n = 3).

The single cell dimensions were analyzed in triplicate in Fiji® and resulted in a length of 112.99 ± 5.29 µm. The videos acquired were analyzed in parallel with the three methods described above and displayed in Fig. 3. Overall, the estimated kinematics were not different using MM or CW. On the other hand, ViKiE estimated longer beat duration at 1 Hz of pacing as well as higher times to reach both contraction peaks (t[θ_c_]) at both 1 and 2 Hz and relaxation peaks (t[θ_r_]) at 1 Hz pacing (Fig. 3 (c,d)). In detail, the time to reach the relaxation peak was estimated two-fold higher at 1 Hz of pacing. Conversely, the distance between peaks (t[θ_c-r_]) did not change within the methods. Two-way ANOVA was performed to detect the frequency pacing effect, but not displayed in Fig. 3 for an easier graphical representation. CW detected a difference in the beat duration between 0.5 Hz and 1 Hz (p = 0.0031), whilst MM highlighted a decreasing difference within t[θ_c-r_] at 1 and 2 Hz (p = 0.0392). ViKiE disclosed a significant increase in the t[θ_c_] between 0.5 Hz and 1 Hz (p = 0.0002) as well as an indicative decrease between the time to reach the peak of contraction (t[θ_c_]) among 1 Hz and 2 Hz (p = 0.0138). Lastly, we observed a significant difference between the t[θr] in the ViKiE evaluation between 0.5 Hz and 1 Hz (p < 0.0001) and 1 Hz and 2 Hz (p < 0.0001).

**Figure 4:**
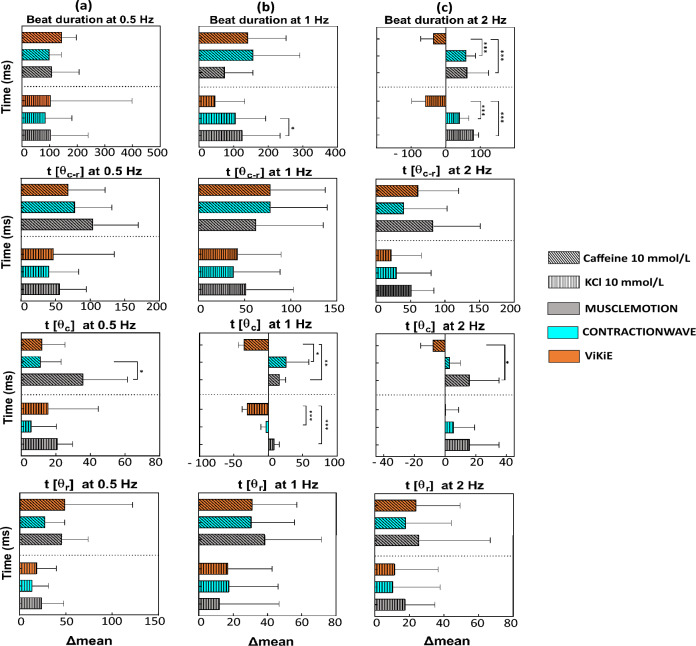
Kinematic analysis of single cells treated with caffeine and KCl, normalized for the control mean value. Vertical-themed bars, 10 mmol/L KCl effect. Oblique-themed bars, 10 mmol/L caffeine-treated cells. X-axis: differences of mean values of the parameters. Grey: MM, Cyan: CW, Orange: ViKiE. (**a**) The four parameters measured at 0.5 Hz pacing frequency. (**b**) Parameters assessed at 1 Hz pacing. (**c**) The same measurements at 2 Hz. Two-way ANOVA was performed with Tukey statistical hypothesis for multiple comparison, significance levels defined *p < 0.05, **p < 0.01, ***p < 0.001 (n = 3).

 As we observed that the three open-source software programs were able to reliably estimate the cell movement, we further tested their sensibility after the administration of KCl and caffeine. In Fig. 4, the differences in time between the untreated and treated conditions are reported. Regarding the hyperkalemia effects (Fig. 4, vertical-themed bars), the three software programs agreed overall on the effects of the treatment, except for the t[θc] at 1 Hz where both CW and ViKiE estimated a shortening in time of contraction (− 3.60 ms for CW, − 30.98 ms for ViKiE and 8.73 ms for MM) (Fig. 4b). Additionally, ViKiE detected a shortening in the beat duration at 2 Hz, in contrast with CW and MM measures (− 44.57 ms for ViKiE versus 30.41 ms for CW and 61.60 ms for MM) (Fig. 4c).

The caffeine effects (Fig. 4, oblique-themed bars) were detected with consistency between the three methods with only few exceptions. In detail, the t[θc] at 1 Hz and 2 Hz showed an opposite trend for ViKiE (− 24 ms vs 52.82 ms for CW and 33.51 ms for MM), as well as the beat duration at 2 Hz (− 27.62 ms for ViKiE versus 45.11 ms for CW and 46.81 ms for MM). As mentioned, despite few differences possibly due to different tracking principles, the three methods evaluated the same effects. To further highlight differences, we calculated and compared the ratios between the mean values at 1 and 2 Hz over 0.5 Hz for each tracking method and observed that ViKiE denoted higher values compared to the other two software programs. In detail, the beat duration at 1 Hz/0.5 Hz (Fig. 4a) resulted in a ratio greater than the unit, in contrast to MM and CW (1.06 vs 0.94 and 0.73, respectively). Moreover, when measuring the time to maximum contraction speed, ViKiE had the same trend as the other two software programs but with higher ratios, thus confirming major sensitivity (1 Hz/0.5 Hz: 1.53 vs 1.19 and 1.10; 2 Hz/0.5 Hz: 1.19 vs 1.11 and 1.08). Lastly, considering the t[θ_r_], ViKiE was in accordance with MM but detected a ratio of 2.26 compared to 1.04. In summary, in untreated cells, the three methods had a good temporal correspondence when estimating the peak-to-peak distance (t[θ_c-r_]) despite the frequencies applied to the cells.

### 3D structure results

**Figure 5:**
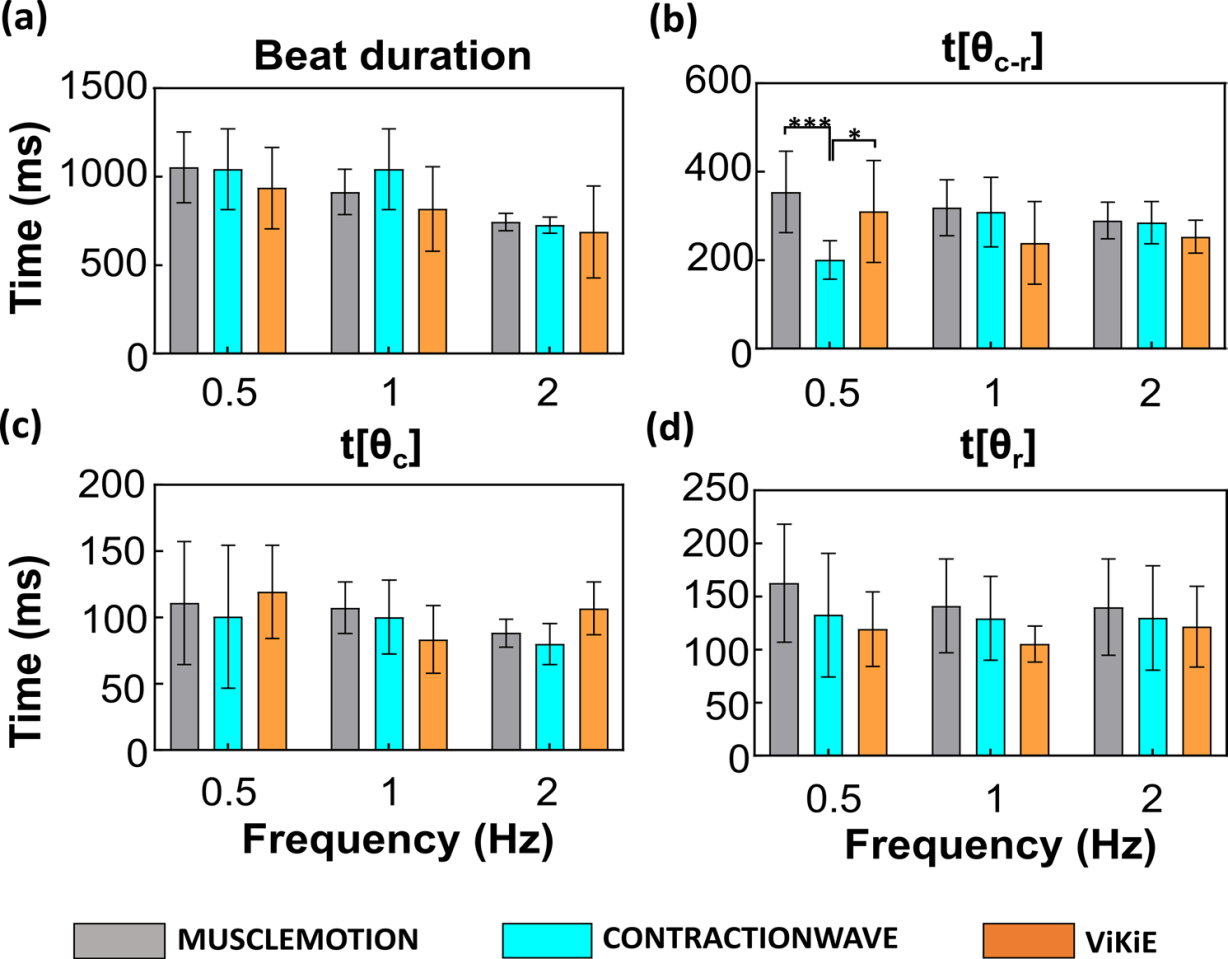
Frequency response of cardioids measured with the three open-source software programs. Grey: MM, Cyan: CW, Orange: ViKiE. X-axis: the frequency of stimulation in Hz (s^−1^). Y-axis: time occurred in milliseconds. (**a**) Beat duration of the untreated cardioids for each algorithm. (**b**) The estimation of the time occurring between the speed peaks at both contraction and relaxation. (**c**) The maximum speed reached during the contraction phase. (**d**) The maximum speed reached during the relaxation phase. Two-way ANOVA was performed with Tukey statistical hypothesis for multiple comparisons, with significance levels defined *p < 0.05, **p < 0.01, ***p < 0.001 (n = 3).

The cardioid dimensions were analyzed in triplicates in Fiji® and resulted in a diameter of 454.13 ± 16.91 µm. As mentioned, the maximum pacing frequency to keep the 1:1 ratio was 1 Hz for the cardioids, therefore we adopted the same kinematic analysis performed on single cells but in following pacing steps: 0.5 Hz, 0.75 Hz and 1 Hz. The results reported in Fig. 5 showed no significant differences between the three software programs at the increasing frequency of stimulation except for t[θc-r], where we observed a nearly two-fold difference between CW and the other two (Fig. 5b). In Fig. 5, the frequency response highlighted an overall coherent estimation of the parameters throughout the protocol and within methods.

**Figure 6:**
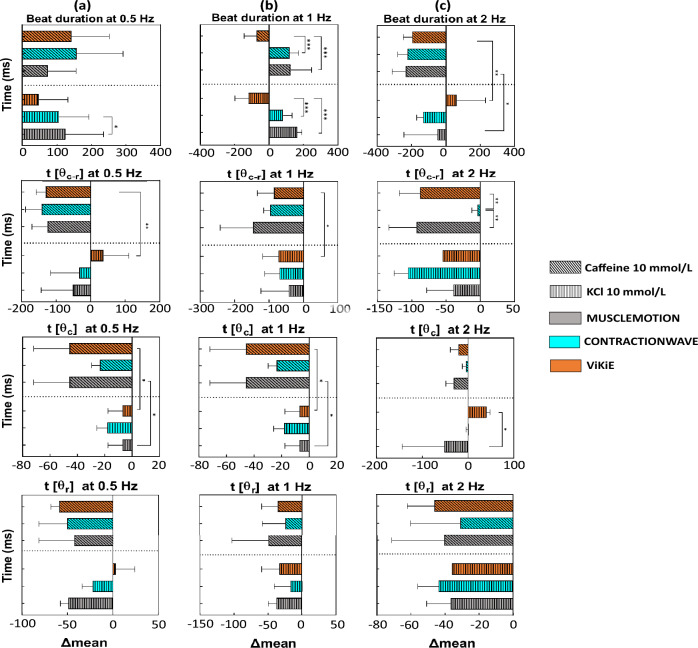
Kinematic analysis of cardioids treated with caffeine and KCl, normalized with the control mean value. Vertical-themed bars, 10 mmol/L KCl effect. Oblique-themed bars, 10 mmol/L caffeine-treated cells. Grey: MM, Cyan: CW, Orange: ViKiE. (**a**) All the kinematic parameters evaluated at 0.5 Hz pacing, (**b**) all the parameters evaluated with cardioids paced at 0.75 Hz. (**c**) Parameters measured at 1 Hz paced cardioids (n = 3).

Then, we assessed the sensitivity of the three methods after the administration of caffeine and KCl as previously performed on cardioids (Fig. 6, vertical-themed histograms). The results are reported as the difference in time between the untreated condition and the treatment. Concerning the caffeine effect (Fig. 6, oblique-themed histograms), the same trend can be appreciated within the software even if a minor but not significant differences can be observed (Fig. 6a). In particular, ViKiE and MM showed a good temporal correlation, estimating all parameters within the same time range. When the treatment switched to KCl (Fig. 6, vertical-themed histograms), ViKiE detected a prolongation of the four kinematic parameters at 0.5 Hz; for instance, beat duration estimated of 62.22 ms versus − 133.1 ms and − 49.39 ms from CW and MM respectively (Fig. 6a).

Both MM and ViKiE managed to discern different kinematic effects of the two treatments in all the parameters. The time of the relaxation peak was the only parameter without statistical differences. To further investigate the difference in sensitivity between the three software programs, the 0.75 Hz/0.5 Hz and 1 Hz/0.5 Hz ratios were calculated as previously described in single cells. Interestingly, MM and ViKiE estimated consistent values in these ratios for both beat duration and t[θ_c-r_]. When analyzing differences in t[θc], ViKiE discriminated a major reduction in the 1 Hz/0.5 Hz ratio (0.70 vs 0.97 of MM), whilst the 0.75 Hz/0.5 Hz ratio was almost comparable between the two software programs (0.89 of ViKiE vs 0.80 of MM). Lastly, MM showed higher sensitivity when discriminating the t[θ_r_] ratio values compared to both CW and ViKiE (0.75 Hz/0.5 Hz: 0.87 vs 0.97 of CW and 0.88 of ViKiE; 1 Hz/0.5 Hz: 0.86 vs 0.98 of CW and 1.02 of ViKiE). Concerning CW sensitivity, its estimations were mostly in line with MM except for t[θc-r] which displayed high ratios (0.75 Hz/0.5 Hz: 1.54 and 1 Hz/0.5 Hz:1.42) compared to the shorter values of both MM and ViKiE (ratios smaller than 1).

### Machine learning results

**Figure 7:**
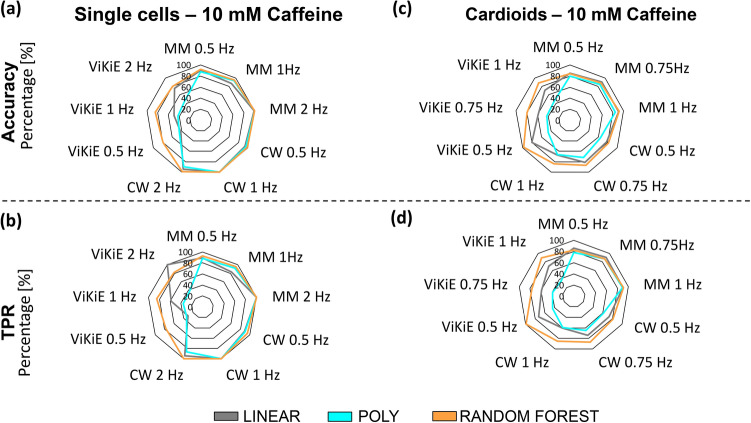
Comparison of performance metrics (Accuracy and TPR) using three different machine learning (ML) algorithms. (**a**) and (**b**) ML was applied on single beat parameters from three motion tracking software (MUSCLEMOTION, CONTRACTIONWAVE and ViKiE) in single cells, at the following stimulation frequencies (0.5 Hz, 1 Hz, 2 Hz), and after 10 mmol/L caffeine administration. (**c**) and (**d**): same as (**a**) and (**b**) but for cardiac spheroids at the following stimulation frequencies (0.5 Hz, 0.75 Hz, 1 Hz). TPR true positive rate. Grey: support vector machine (linear kernel), Cyan: support vector machine (polynomial kernel), Orange: random forest.

**Figure 8:**
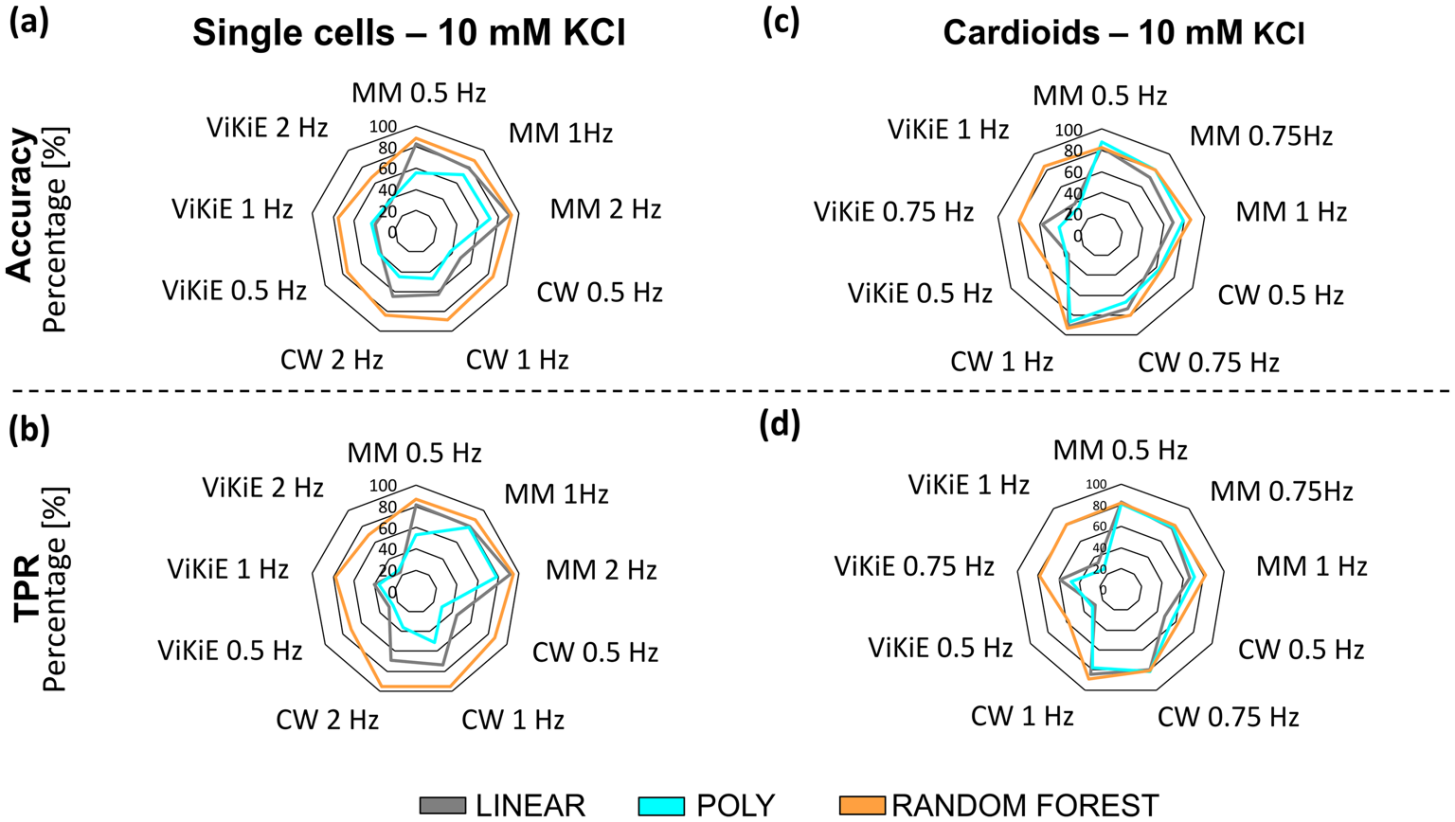
Comparison of performance metrics (Accuracy and TPR) using three different machine learning (ML) algorithms. (**a**) and (**b**) ML was applied on single beat parameters from three motion tracking software (MUSCLEMOTION, CONTRACTIONWAVE and ViKiE) in single cells, at the following stimulation frequencies (0.5 Hz, 1 Hz, 2 Hz), and after 10 mmol/L KCl administration. (**c**) and (**d**): same as (**a**) and (**b**) but for cardiac spheroids at the following stimulation frequencies (0.5 Hz, 0.75 Hz, 1 Hz). TPR true positive rate. Grey: support vector machine (linear kernel), Cyan: support vector machine (polynomial kernel), Orange: random forest.

ML was applied on our dataset to further assess the sensitivity of the three software programs. For this purpose, RF and SVM were used to classify the beats of both adult single ventricular cardiomyocytes and cardioids after treatment with caffeine and KCl. The accuracy and TPR of both RF and SVM applied to the single cells treated with 10 mmol/L caffeine are calculated and reported on radar plots in Fig. 7. SVM, with both linear and polynomial kernels, and RF performed well with both CW and MM training datasets (TPR and accuracy > 90%). On the contrary, ViKiE underperformed with SVM (TPR and accuracy < 60%) but not with RF (accuracy and TPR comprised between 69 and 86%). In Fig. 8, it is possible to observe the performance metrics radar plots of the machine-learning techniques applied to the cardioids treated with 10 mmol/L caffeine. All the classification models performed well with MM compared to CW and ViKiE (TPR and accuracy > 77%). SVM with polynomial kernel was generally underperforming, especially when trained with CW and ViKiE data. Interestingly, the classification model performances for these two software programs were comparable in cardioids. In single cells treated with 10 mmol/L KCl (Fig. 8a,b), RF performed well with MM (TPR > 86%, accuracy > 87%) and CW (TPR > 86%, accuracy > 83%) for all stimulation frequencies. On the contrary, SVM underperformed with ViKiE (TPR and accuracy < 41%). In cardioids treated with 10 mmol/L KCl (Fig. 8 c,d), the ML algorithms classified MM better than the other software programs (TPR and accuracy > 66%), whereas they showed the worst performance with ViKiE. In particular, SVM showed the worst performance with ViKiE (TPR and accuracy < 60%).

To summarize, we observed that at higher frequencies (2 Hz for single cells and 1 Hz for cardioids), RF showed the best performance in both treatments (accuracy > 83%). Overall, ML algorithms better classified MM compared to the other two software programs.

## Discussion

The necessity of evaluating cell movement in a reliable and efficient way has never been so compelling to estimate and discriminate different kinematic properties, especially when it comes to cardiac research applications^35^. Well-established systems, such as IonOptix^®^, do not allow scalability to 3D samples yet adding this limitation to the high economic cost. In this work, we illustrated and compared several open-source methods found in literature which rely on different algorithms for tracking kinematics^18,36,39^. The three software programs base their evaluation on different hypotheses, namely pixel intensity track changes (MM), optical flow analysis (CW), and blob analysis (ViKiE). In this study, *in-silico* preliminary comparison in terms of absolute speed profiles and by four different parameters using two different *in-vitro* models was performed, as illustrated in Fig. 1.

The *in-silico* experiment allowed a preliminary comparison of the three software programs over a ground truth dataset and with known output^36^. The videos were considered as if they were used to establish and validate the linearity of MM output, thus the authors have considered this analysis as a logical starting point. Comparing the results reported in Fig. 2a,b, it is clear how CW showed a more stable performance when analyzing periodically patterned samples, as in isolated single cardiomyocytes. ViKiE, on the other hand, once the trackers were positioned over representative pixel regions, allowed to estimate local movement reliably and with stable output.

The results confirmed the possibility to compare these three software programs over temporal-derived characteristics and assuming MM as a benchmark. Following these results, the work moved towards *in-vitro* models to assess the software in terms of flexibility.

The first model was the single ventricular cardiomyocytes isolated from adult mice which is the most used in *in-vitro* application. We aimed to determine whether the three software programs were temporally detecting the same phenomena and converging on the same paced kinematics. Our results showed that the three software programs had a good temporal correlation when estimating the time to the peak of contraction and relaxation throughout the frequency protocol (cf. Fig. 3b). Conversely, differences emerged in the other three kinematic measurements (cf. Fig. 3a,c,d) suggesting that ViKiE tracking method might have higher sensitivity. Indeed, especially when computing the t[θ_c_] and t[θ_r_], ViKiE showed higher sensitivity in estimating untreated single cells kinematics. The reason may be attributed to the local kinematic evaluation rather than the global computation performed by the other two software programs. However, ViKiE-derived kinematics were a reliable measure as demonstrated by the overall trend compared to the other two methods. The underlined difference was more prominent when the samples were treated with KCl and caffeine (Fig. 4). This is especially true when computing the beat duration at 2 Hz and the t[θ_c_] at 1 Hz and 2 Hz, where ViKiE discriminates the different effects of the treatments and in contrast to both MM and CW measurements (Fig. 4b third panel; Fig. 4c, first and third panels). Overall, our results demonstrated the reproducibility and the temporal correlation between these three tracking algorithms with a few exceptions which will be further discussed.

Cardioids were the second *in-vitro* model employed in this work. This choice was mainly guided by its growing interest for the high-throughput, standardized output^50–52,70,71^. The same analysis pipeline applied to the single cells was adopted to the cardioids, stimulating the samples at 0.5, 0.75 and 1 Hz. From an analytical point of view, CW failed at processing cardioids, even after an image crop was applied to decrease the noise, making it difficult for the user to produce reliable data. Nonetheless, when analyzing the frequency-paced samples, the three software programs agreed on both trend and time scale of the kinematic phenomenon, with the exception for t[θ_c-r_] at 0.5 Hz (Fig. 5b). When calculating the ratios of the parameters at 0.75 Hz/0.5 Hz and 1 Hz/0.5 Hz, MM showed higher sensitivity when discriminating the t[θ_r_] changes. On the other hand, CW sensitivity was aligned with MM except for t[θ_c-r_]. Lastly, MM and ViKiE estimated consistent values in the beat duration ratio as well as the t[θ_c-r_]. The authors speculate that the underperformance of CW over this type of sample may be connected to the performance observed in Fig. 2a. In fact, the spheroid is a highly contrasted object over the background, as the *in-silico* cardiomyocyte of the first dataset (Fig. 2a).

A separate discussion must be made about the treated samples. Once again, the kinematics at 0.5 Hz suggested a higher sensitivity for ViKiE than the other two software programs (Fig. 6a, from the first to the fourth panel), whilst at both 0.75 and 1 Hz ViKiE and MM display the same trend. Specifically, MM analysis reported two opposite effects of 10 mmol/L KCl for both beat duration (Fig. 6b, first panel) and t[θ_c_] (Fig. 6b,c third panels), which may suggest a satisfactory sensitivity for this software in detecting differences. On the other hand, ViKiE outperformed in discriminating the t[θ_c-r_] at 0.5 Hz pacing. The authors speculate that the reason of such varied outcomes of the contractility parameters might lie in the intrinsic characteristics of the tracking software. Overall, when treating the sample, the local property (detected by Vi.Ki.E.) might be highlighting a different, and even opposite, trend compared to the mean behaviour evaluated (MUSCLEMOTION and CONTRACTIONWAVE). This intrinsic difference might be less marked with constructs as cardioids (Fig. 6) compared to the single cells (Fig. 7). The latter are heavily more sensitive compared to 3D construct to environmental triggers, as frequency and inotropic substances, by trivial reason. Moreover, to the best of our knowledge, this constitutes one of the first kinematic studies on treated cardioids, as well as the first application of CW system on this *in-vitro* model.

Concerning the ML analysis, RF outperformed the SVM algorithms, since it did not classify the data points through a limited hyperplane. The algorithm used uncorrelated features, hence, there was an automatic selection of the most relevant ones. SVM was not able to filter the correlated features that could influence the classification negatively. Overall, MM produced parameter-wise more robust data than the other two techniques. Thus, the classification of this software was higher in accuracy and TPR independently from the stimulation frequency. On the other hand, the ML algorithms underperformed while trained with ViKiE dataset due to more sensitive and thus less robust parameters. CW was a good compromise between parameter-wise robustness and machine learning performance.

Hereby is reported a summary table of the main characteristics of the software used on two of the most used *in-vitro* standards (Table 2). This work aimed to determine the reliability of the tracking methods compromising between the time effort and computational robustness. One of the main results of this study was to demonstrate that not all the methods are robust when applied to different models. This evidence constitutes a challenge for developers to create an all-in-one algorithm that must satisfy the most general experimental conditions and at the same time be reliable and user-friendly. These listed characteristics are reported in Table 2, hoping to serve as a guideline for researchers approaching cell kinematics tracking.

**Table 2:** Summary of the software characteristics.

	MUSCLEMOTION	CONTRACTIONWAVE	ViKiE
Input files extension	.tiff, .png	.avi, .tiff, .png	.avi, .tiff, .png
Code repository	Yes (Fiji^®^)	Yes (Python)	Yes, on request (MATLAB®)
User-friendly	Yes	Yes	Yes, requires basic MATLAB knowledge
Time for analysis	Medium	High, but parallelized analysis	High
Scalability	Yes	No	Yes
Sensitivity	High in cardioids	Medium	High in cardioids and single cells
Robustness	High	Medium	Depending on motion tracker software used
Units of measures	Average movement in arbitrary units (a.u.)	Average movement (μm/s)	Local movement (μm/s)
Predictive accuracy (random forest)	Accuracy > 87%	Accuracy > 83%	Accuracy > 79%

The input files extension refers to the format read by the program/pipeline. The importance of having the original code allows the user to customize the program according to experimental necessities. The user-friendliness factor is essential for an intuitive interface and related documentation might improve user experience. When analyzing memory-consuming video, the time required to process the data may depend on the type of algorithm performed. The ability of the software to correctly detect movement from different biological samples is essential to determine its scalability. Through machine learning-aided analysis, we have demonstrated the overall sensitivity and robustness of the three methods with MUSCLEMOTION outperforming the other software programs. Lastly, the type and units of measures computed by the software programs, whether they calculate the average or local movement as well as if the output is displayed in either arbitrary or physical units. Predictive accuracy of the machine learning performed on the dataset derived from the three software is reported to complete this comprehensive comparison.

In conclusion, the present study reviewed three open-source software programs and tested their performances in reliability and scalability. Even though the algorithms based their kinematic evaluations on a different physical basis, we were able to temporally compare them and appreciate their consistency in both our *in-vitro* models. Overall, ViKiE tends to be more sensitive because of the local, rather than global, kinematic measure, and may constitute an advantage when the biological sample does not have isovolumetric contraction. On the other hand, both MM and CW showed a temporal correlation, having the advantage of a global evaluation while executing a timesaving analysis.

An improvement in the study could be the integration and monitoring of the wash-out phase of the substances through a microfluidic system. The ability to measure not only the speed of contraction but also the contraction force could provide more insightful data. Furthermore, as ViKiE is based on different software pipeline, an improvement could be the use of another tracking method different from Video Spot Tracker to guarantee a more robust dataset and thus a better classification performance. Of course, for the ML training, an increased number of data would be beneficial for the correct prediction outcome.

### Supplementary Information

Supplementary Information.

## Data Availability

The datasets generated during and/or analysed during the current study are available from the corresponding author on reasonable request.
