# Supplementary material for: Unlocking cardiac motion: assessing software and machine learning for single-cell and cardioid kinematic insights
Source: Sci Rep. 2024 Jan 20;14:1782. doi: 10.1038/s41598-024-52081-9 (PMC10799933; doi:10.1038/s41598-024-52081-9)
Supplement: Supplementary file 1 — Supplementary Information. [file 41598_2024_52081_MOESM1_ESM.pdf]

Unlocking Cardiac motion: Assessing Software and Machine Learning for Single-Cell and Cardioid Kinematic Insights

Margherita Burattini[1,2], Francesco Paolo Lo Muzio[2,3], Mirko Hu[2], Flavia Bonalumi[2], Stefano Rossi[2], Christina Pagiatakis[4,5], Nicolò Salvarani[4,6], Lorenzo Fassina[7], Giovanni Battista Luciani[1], Michele Miragoli[*2,4]

1) University of Verona, Department of Surgery, Dentistry and Maternity, Verona, Italy

2) University of Parma, Department of Medicine and Surgery, Parma, Italy

3) Deutsches Herzzentrum der Charité, Department of Cardiology, Angiology and Intensive Care Medicine, Berlin, Germany

4) Humanitas Research Hospital — IRCCS, Rozzano (Milan), Italy

5) University of Insubria, Department of Biotechnology and Life Sciences, Varese, Italy

6) Institute of Genetic and Biomedical Research (IRGB), UOS of Milan, National Research Council of Italy , Milan, Italy

7) University of Pavia, Department of Electrical, Computer and Biomedical Engineering, Pavia, Italy

SUPPLEMENTARY INFORMATION

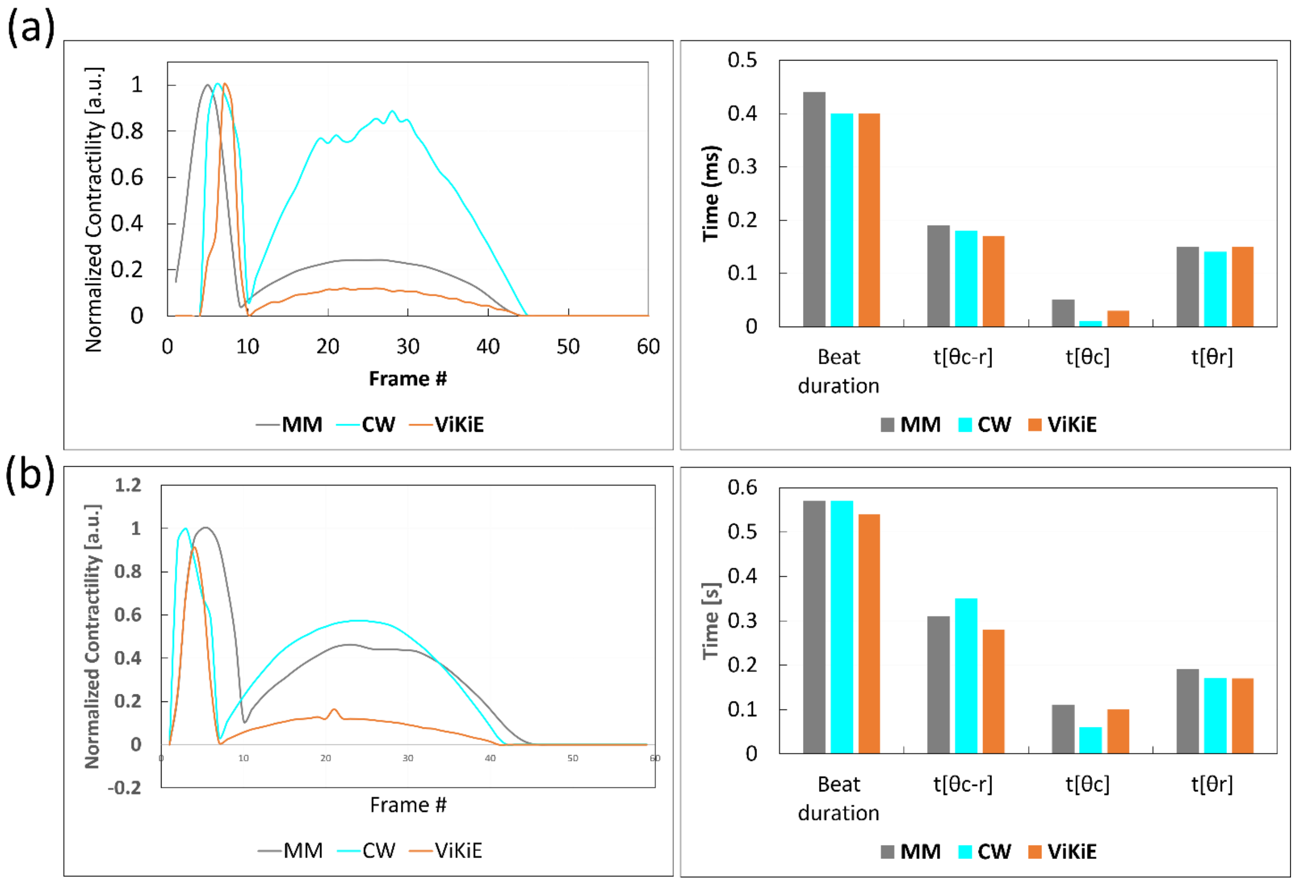

Fig. S1 Comparison of the three open-source software programs with in-silico cardiomyocytes with 50% time-to-peak. All the profiles are reported as normalized and in absolute values. a) The first experiment with “grey” patterned cardiomyocyte. The grey profile is the benchmark considered in this study (MUSCLEMOTION), the cyan profile derives from CONTRACTIONWAVE evaluation, and the orange profile is the ViKiE estimation. The histograms on the right side represent the four parameters extracted by the profiles and measured by the three methodologies. b) The second experiment with “fog” patterned cardiomyocyte. As in panel a), the profiles and histograms of the software evaluations are reported to address the ground-truth comparison.

	Frequency (Hz)	TPR			Accuracy			F1-score		
		SVM (linear)	SVM (poly)	Random Forest	SVM (linear)	SVM (poly)	Random Forest	SVM (linear)	SVM (poly)	Random Forest
**MUSCLEMOTION**	0.5	89.17 ± 14.38	88.00 ± 16.33	93.00 ± 12.82	90.25 ± 8.31	88.00 ± 8.61	92.25 ± 7.26	84.36 ± 14.47	79.62 ± 16.27	86.46 ± 13.68
	1	82.82 ± 8.93	92.65 ± 6.61	95.20 ± 5.51	87.00 ± 5.47	93.75 ± 3.92	95.25 ± 3.64	87.18 ± 6.16	93.08 ± 5.12	94.78 ± 4.75
	2	100.00 ± 0.00	100.00 ± 0.00	100.00 ± 0.00	99.40 ± 1.17	99.93 ± 0.33	99.93 ± 0.33	99.38 ± 1.17	99.93 ± 0.33	99.95 ± 0.26
**CONTRACTIONWAVE**	0.5	88.42 ± 13.76	90.08 ± 12.28	95.00 ± 10.95	93.75 ± 5.72	95.75 ± 4.72	98.50 ± 3.47	87.74 ± 13.28	91.38 ± 11.40	93.68 ± 11.28
	1	100.00 ± 0.00	100.00 ± 0.00	100.00 ± 0.00	100.00 ± 0.00	100.00 ± 0.00	100.00 ± 0.00	100.00 ± 0.00	100.00 ± 0.00	100.00 ± 0.00
	2	94.34 ± 4.07	86.24 ± 6.25	100.00 ± 0.00	94.75 ± 2.83	90.06 ± 3.95	99.19 ± 1.38	94.76 ± 2.79	90.67 ± 3.70	99.13 ± 1.50
**ViKiE**	0.5	31.00 ± 17.98	33.58 ± 14.92	77.00 ± 18.02	42.50 ± 13.12	45.25 ± 12.27	79.75 ± 10.16	32.38 ± 17.15	40.95 ± 16.73	71.19 ± 16.80
	1	58.46 ± 17.10	35.66 ± 10.92	85.41 ± 9.12	49.13 ± 8.20	42.63 ± 7.00	84.00 ± 6.35	45.98 ± 10.14	48.20 ± 13.84	81.96 ± 7.82
	2	100.00 ± 0.00	37.31 ± 18.83	81.25 ± 7.41	75.00 ± 6.15	45.67 ± 6.00	80.33 ± 5.08	65.44 ± 8.30	29.87 ± 13.27	79.19 ± 5.98

Table S1 Summary of the MACHINE LEARNING PERFORMANCES – SINGLE CELLS Analysis related to single cells exposed to caffeine at 10 mmol/L. The columns report in order true positive rate (TPR), accuracy and Fisher score (F1 score). The rows, grouped by each software, list the performances of the different ML models over the different stimuli operated on the samples. The data were used to produce the radar plot reported on Figure S3. TPR: true positive rate, SVM: support vector machine. The data are reported as mean±standard deviation.

	Frequency (Hz)	TPR			Accuracy			F1-score		
		SVM (linear)	SVM (poly)	Random Forest	SVM (linear)	SVM (poly)	Random Forest	SVM (linear)	SVM (poly)	Random Forest
**MUSCLEMOTION**	0.5	86.22 ± 6.59	77.92 ± 7.46	82.02 ± 7.46	85.57 ± 4.12	79.50 ± 5.19	84.36 ± 5.12	84.77 ± 5.20	79.99 ± 5.73	84.22 ± 5.65
	1	90.90 ± 4.91	86.85 ± 5.50	86.99 ± 5.63	90.67 ± 3.68	84.89 ± 4.20	88.11 ± 3.18	90.42 ± 3.76	83.97 ± 4.66	88.07 ± 3.47
	2	90.14 ± 4.17	89.45 ± 4.79	90.97 ± 4.24	90.29 ± 2.96	84.58 ± 3.66	91.92 ± 2.58	90.20 ± 3.04	83.45 ± 4.30	91.98 ± 2.60
**CONTRACTIONWAVE**	0.5	73.57 ± 6.82	60.92 ± 7.15	81.55 ± 7.43	77.79 ± 4.84	64.71 ± 5.69	82.07 ± 4.78	79.42 ± 5.04	70.36 ± 5.80	81.58 ± 5.99
	1	73.88 ± 7.61	64.62 ± 6.89	86.83 ± 7.00	81.50 ± 5.85	72.08 ± 5.50	86.58 ± 4.70	84.01 ± 5.35	77.64 ± 5.28	86.46 ± 5.01
	2	60.71 ± 5.91	59.40 ± 5.55	85.08 ± 5.46	66.83 ± 4.85	66.00 ± 4.73	83.83 ± 4.08	73.65 ± 4.52	73.71 ± 4.43	83.09 ± 4.25
**ViKiE**	0.5	73.04 ± 7.76	44.03 ± 14.55	98.90 ± 1.88	79.86 ± 5.77	45.57 ± 5.78	97.64 ± 1.90	82.56 ± 5.52	39.31 ± 12.81	97.44 ± 2.10
	1	59.31 ± 8.15	38.49 ± 14.05	76.59 ± 6.71	58.95 ± 6.35	42.84 ± 3.92	80.00 ± 4.41	59.18 ± 8.69	30.63 ± 11.65	81.39 ± 4.09
	2	70.04 ± 8.25	35.55 ± 11.57	89.94 ± 4.63	70.54 ± 5.98	44.43 ± 3.26	88.54 ± 2.83	74.02 ± 5.80	37.33 ± 13.38	88.49 ± 2.87

Table S2 Summary of the machine learning performances – cardioids Analysis related to cardioids exposed to caffeine at 10 mmol/L. The columns report in order true positive rate (TPR), accuracy and Fisher score (F1 score). The rows, grouped by each software, list the performances of the different ML models over the different stimuli operated on the samples. The data were used to produce the radar plot reported on Figure S3. TPR: true positive rate, SVM: support vector machine. The data are reported as mean±standard deviation.

	Frequency (Hz)	TPR			Accuracy			F1-score		
		SVM (linear)	SVM (poly)	Random Forest	SVM (linear)	SVM (poly)	Random Forest	SVM (linear)	SVM (poly)	Random Forest
MUSCLEMOTION	0.5	81.83 ± 17.26	53.00 ± 23.30	86.83 ± 13.46	83.25 ± 9.41	56.00 ± 11.67	88.25 ± 8.04	73.92 ± 16.12	39.13 ± 17.01	84.37 ± 12.50
	1	80.12 ± 10.96	78.27 ± 13.22	87.80 ± 8.14	78.75 ± 7.41	70.00 ± 7.95	87.25 ± 5.55	76.14 ± 9.56	63.28 ± 11.22	86.20 ± 6.44
	2	91.42 ± 5.19	78.47 ± 9.09	93.96 ± 4.48	90.40 ± 3.89	71.67 ± 5.13	92.33 ± 3.56	89.34 ± 4.79	66.61 ± 7.25	91.66 ± 3.94
CONTRACTIONWAVE	0.5	44.75 ± 20.54	29.08 ± 16.87	86.83 ± 15.32	48.75 ± 13.10	37.75 ± 11.31	85.00 ± 8.14	39.07 ± 16.44	29.73 ± 14.82	77.03 ± 14.56
	1	74.02 ± 16.90	50.87 ± 21.01	95.23 ± 5.63	62.56 ± 9.36	47.00 ± 7.32	88.56 ± 5.21	55.03 ± 12.07	33.05 ± 12.00	86.60 ± 6.81
	2	69.03 ± 8.92	35.74 ± 13.30	95.70 ± 4.30	64.75 ± 5.85	45.06 ± 5.07	83.81 ± 4.95	63.01 ± 6.30	37.94 ± 13.06	81.49 ± 5.80
ViKiE	0.5	28.92 ± 16.61	25.50 ± 15.48	70.75 ± 18.33	38.75 ± 11.15	40.50 ± 9.86	75.25 ± 10.43	29.10 ± 14.70	29.11 ± 15.35	67.30 ± 16.63
	1	40.25 ± 9.71	35.93 ± 10.99	77.71 ± 12.12	39.38 ± 7.24	43.25 ± 7.07	74.88 ± 6.91	47.28 ± 10.33	48.45 ± 13.89	70.41 ± 10.26
	2	23.59 ± 9.99	26.00 ± 10.53	69.75 ± 9.72	37.33 ± 4.53	38.17 ± 5.00	66.33 ± 7.10	28.32 ± 12.14	32.64 ± 13.23	64.85 ± 8.29

Table S3 Summary of the machine learning performances –single cells. Analysis related to cardioids exposed to KCl at 10 mmol/L. The columns report in order true positive rate (TPR), accuracy and Fisher score (F1 score). The rows, grouped by each software, list the performances of the different ML models over the different stimuli operated on the samples. The data were used to produce the radar plot reported on Figure S3. TPR: true positive rate, SVM: support vector machine. The data are reported as mean±standard deviation.

	Frequency (Hz)	TPR			Accuracy			F1-score		
		SVM (linear)	SVM (poly)	Random Forest	SVM (linear)	SVM (poly)	Random Forest	SVM (linear)	SVM (poly)	Random Forest
MUSCLEMOTION	0.5	83.05 ± 9.07	81.43 ± 7.03	82.29 ± 7.95	82.73 ± 6.44	87.64 ± 4.59	82.82 ± 4.92	82.44 ± 7.20	88.41 ± 4.75	82.69 ± 5.55
	1	76.20 ± 9.25	77.73 ± 7.72	80.10 ± 8.11	71.45 ± 6.68	80.27 ± 6.30	80.64 ± 5.51	71.04 ± 7.22	81.58 ± 6.54	81.79 ± 5.75
	2	66.51 ± 9.06	70.89 ± 7.67	81.87 ± 7.71	69.00 ± 6.62	79.29 ± 5.75	86.14 ± 4.84	68.64 ± 8.09	80.86 ± 6.17	85.64 ± 5.64
CONTRACTIONWAVE	0.5	48.42 ± 14.69	56.00 ± 12.22	62.80 ± 13.28	55.67 ± 11.80	63.50 ± 10.77	65.17 ± 8.71	55.46 ± 15.21	67.56 ± 12.16	64.11 ± 11.30
	1	79.66 ± 10.16	81.39 ± 12.38	80.67 ± 9.01	73.82 ± 6.04	67.45 ± 6.20	79.82 ± 5.50	72.40 ± 7.61	61.99 ± 8.63	80.37 ± 5.65
	2	84.60 ± 12.71	77.95 ± 13.29	89.00 ± 11.83	91.20 ± 7.15	86.40 ± 8.39	93.60 ± 5.66	88.68 ± 10.63	84.42 ± 11.07	89.85 ± 10.36
ViKiE	0.5	28.28 ± 13.12	31.37 ± 16.96	58.02 ± 16.45	36.17 ± 8.21	38.50 ± 8.76	58.00 ± 10.56	29.49 ± 12.19	26.60 ± 11.65	54.67 ± 13.56
	1	59.06 ± 14.51	47.69 ± 19.97	78.99 ± 10.76	57.25 ± 10.52	41.25 ± 7.67	79.75 ± 7.09	57.08 ± 11.97	33.64 ± 10.97	78.40 ± 8.42
	2	33.18 ± 16.39	25.53 ± 12.78	81.13 ± 15.46	39.00 ± 10.48	34.60 ± 9.08	85.00 ± 7.90	35.01 ± 14.02	31.40 ± 13.71	79.59 ± 13.98

Table S4 Summary of the machine learning performances – cardioids. Analysis related to cardioids exposed to KCl at 10 mmol/L. The columns report in order true positive rate (TPR), accuracy and Fisher score (F1 score). The rows, grouped by each software, list the performances of the different ML models over the different stimuli operated on the samples. The data were used to produce the radar plot reported on Fig. S3. TPR: true positive rate, SVM: support vector machine. The data are reported as mean±standard deviation.
